# Acyl metalloids: conformity and deviation from carbonyl reactivity

**DOI:** 10.1039/d1sc00077b

**Published:** 2021-03-10

**Authors:** Aleksandra Holownia, Chirag N. Apte, Andrei K. Yudin

**Affiliations:** Davenport Laboratories, Department of Chemistry, University of Toronto 80 St. George St. Toronto Ontario M5S 3H6 Canada andrei.yudin@utoronto.ca

## Abstract

Once considered as mere curiosities, acyl metalloids are now recognized for their utility in enabling chemical synthesis. This perspective considers the reactivity displayed by acylboron, -silicon, -germanium, and tellurium species. By highlighting the role of these species in various transformations, we demonstrate how differences between the comprising elements result in varied reaction outcomes. While acylboron compounds are primarily used in polar transformations, germanium and tellurium species have found utility as radical precursors. Applications of acylsilanes are comparatively more diverse, owing to the possibility to access both radical and polar chemistry.

## Abstract 2

Originally considered as fundamental curiosities, acyl metalloids have proven to be useful tools in synthesis. This perspective aims to highlight the modes of reactivity accessible to boron, silicon, germanium and tellurium species.

## Graphical abstract

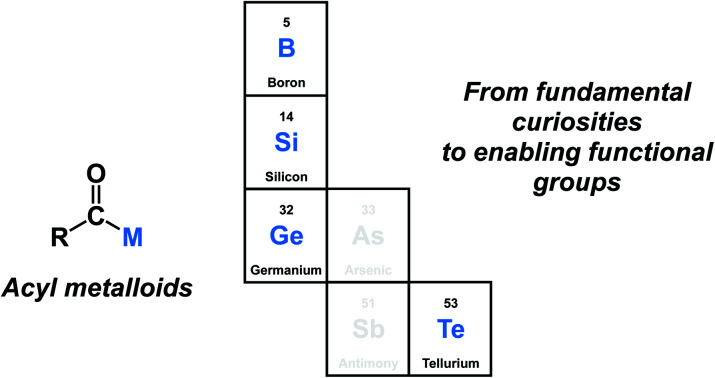

## Introduction

Functional groups (FGs) are atoms or groups of atoms that have similar chemical properties when they appear in different molecules.^1^ The carbonyl group, which characterizes aldehydes, ketones, carboxylic acids, esters, and amides, represents one of the most fundamentally important functionalities in a synthetic chemist's toolbox. This is due to the rich chemistry available to carbonyls, which is largely a consequence of the polarized C^*δ*+^–O^*δ*−^ bond. This polarization dictates nucleophilic attack at the carbon and reactions with electrophiles *via* oxygen. A carbonyl derivative RCOX is also influenced by the X substituent, which can alter the electronic nature of the carbonyl group.

**Fig. 1:**
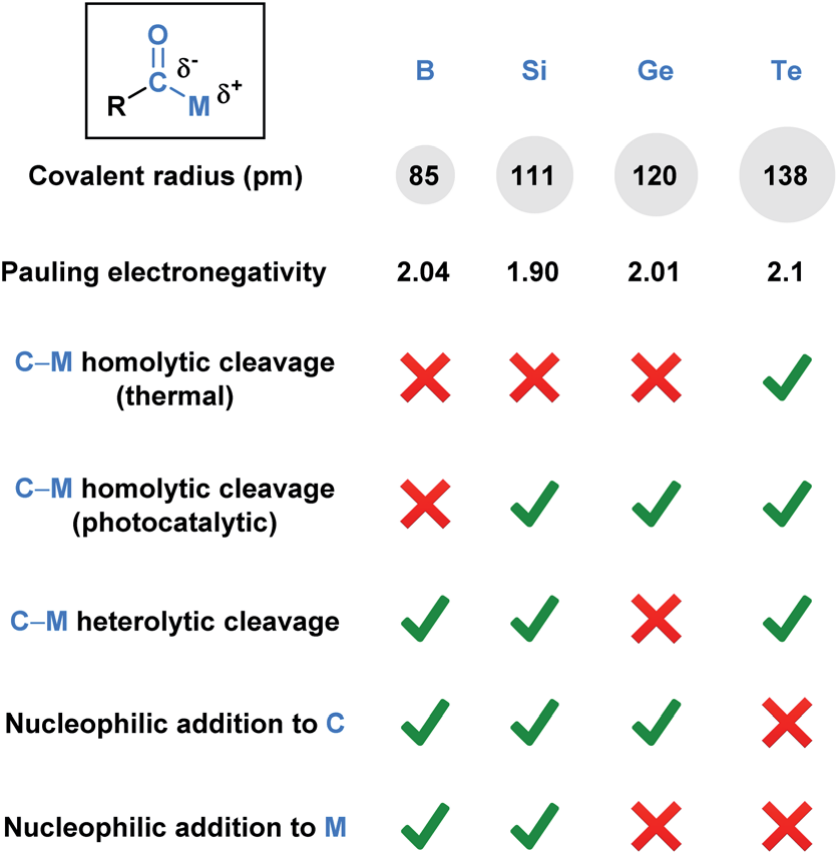
Comparison of the fundamental properties and reported chemical reactivity of acylboron, -silicon, -germanium, and tellurium species.

Acyl metalloids have been increasingly utilized in organic synthesis. They represent an intriguing class of compounds because the carbonyl carbon is more electronegative than the C-bound metalloid, which results in an amphoteric carbon center that is nucleophilic through its carbon-metalloid σ-bond and electrophilic through its carbonyl π-bond. Boron, silicon, germanium, arsenic, antimony and tellurium are the elements commonly recognized as metalloids.^2^ This perspective aims to provide a descriptive overview of the recent literature regarding synthetically useful acyl metalloids, such as boron, silicon, germanium and tellurium species, and to offer a glimpse into the diverse reactivity profiles of acyl metalloids. The reactivity resulting from this combination affords new opportunities to build molecular frameworks, and, in some instances, the potential to transfer the metalloid functionality through carbonyl manipulation. Although there is some overlap between the modes of reactivity, subtle differences between the elements lead to chemical transformations that are unique to the metalloid under consideration. In an effort to categorize these distinctions, the chemical reactivities of each subclass has been summarized (Fig. 1).

## Boron

**Scheme 1:**
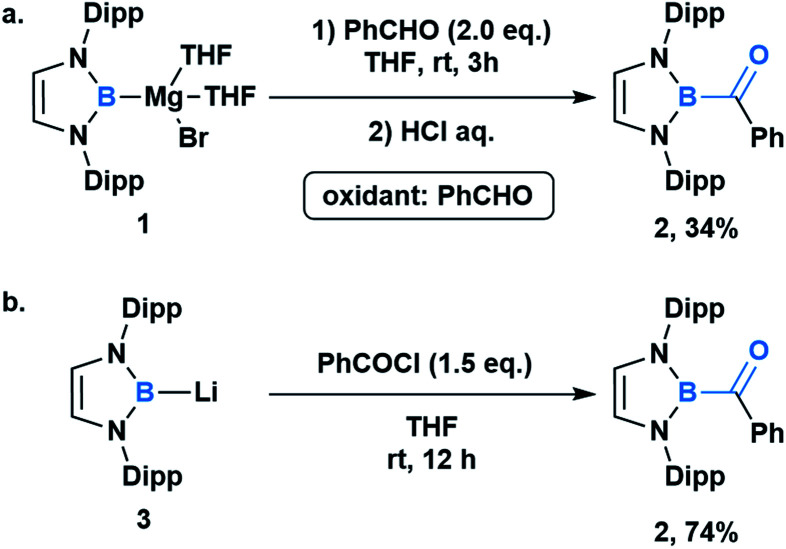
Synthesis and characterization of the first acylboron compound **2**; (a) reaction between borylmagnesium **1** with benz-aldehyde; (b) reaction between borylithium **3** and benzoyl chloride. Dipp = 2,6-(*i*-Pr)_2_C_6_H_3_.

**Fig. 2:**
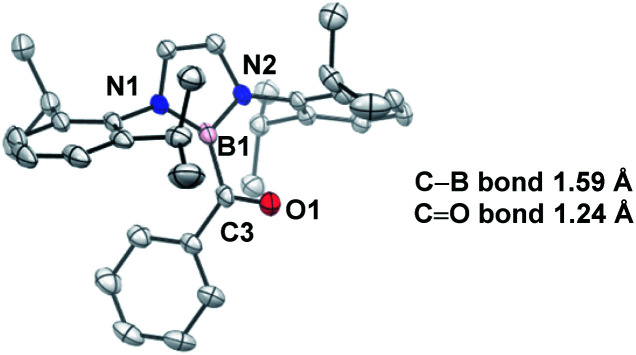
X-ray crystal structure of acylborane **2** contains a C=O bond length which is ketone-like (CCDC#662648). For clarity, hydrogen atoms are omitted. Thermal ellipsoids set at 50% probability.

In comparison to other acyl metalloids, acylboron species had remained elusive until the late 2000s.^3–5^ Initially, these compounds were proposed as intermediates in several transformations, but their isolation and characterization were not realized due to their presumed susceptibility to oxidation and rearrangement.^6–11^ The first example of an isolable acylboron compound was reported by Nozaki and coworkers in 2007, who accessed this class of compounds by reacting borylmagnesium **1** with benzaldehyde (Scheme 1a).^12^ It was later demonstrated that boryllithium **3** could be reacted with benzoyl chloride delivering acylboron in higher yields (Scheme 1b).^13,14^ X-ray crystallographic analysis revealed a C=O bond length of 1.24 Å suggesting that the carbonyl is ketone-like, while the IR frequency of 1618 cm^−1^ is lower than a typical ketone or aldehyde (Fig. 2). The stability of this acylborane can be rationalized by the π-donor ability of the diaminoboryl substituent to the p orbital of the boryl group.

**Fig. 3:**
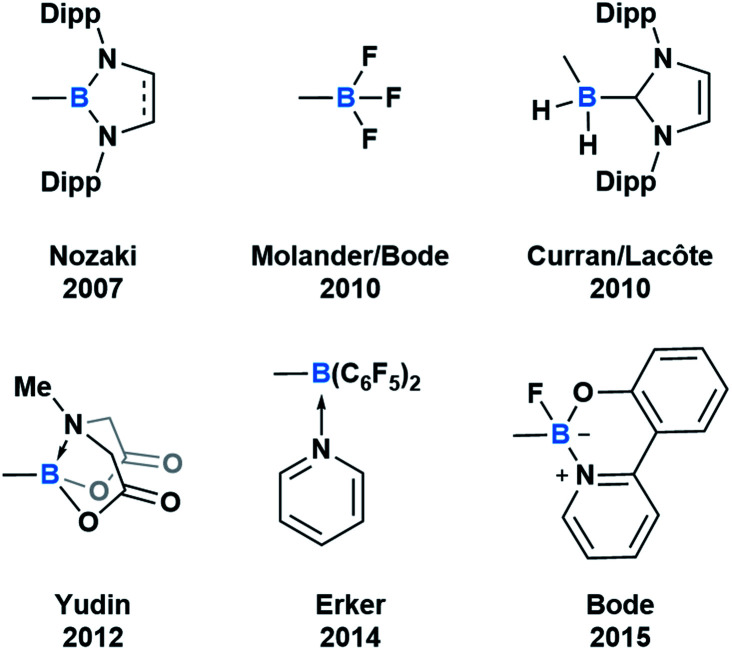
Select examples of fully characterized acyl boron compounds. The group bound to boron plays a significant role in stabilizing boron and influencing reactivity.

Throughout subsequent years, several approaches to generate various acylboron compounds were reported (Fig. 3). Molander demonstrated the first synthesis and isolation of potassium acyltrifluoroborates (KATs) by treating an acyl anion equivalent with triisopropyl borate.^15,16^ Curran and Lecôte reported a novel class of N-heterocyclic carbene stabilized acyl boranes from the reaction of boryl iodide with a lithium reagent.^17^ Yudin developed a Barton–McCombie decarboxylation/Dess–Martin oxidation approach towards the synthetically useful class of *N*-methyliminodiacetic acid (MIDA) acylboronates.^18^ Erker showed that pyridine-stabilized formylborane can be obtained through the 1,1-hydroboration of carbon monoxide with HB(C_6_F_5_)_2_ at an intramolecular frustrated Lewis pair, followed by liberation with pyridine.^19^ Most recently, Bode described an approach to the exceptionally stable monofluoroboronates which can be accessed from the corresponding KATs.^20^ Based on these reported examples, it is evident that the ligand bound to boron significantly influences the capacity to synthesize and isolate acylboron compounds.

To date, applications of acylboron species primarily take advantage of the electrophilic character of the carbonyl functionality, whereby the fate of the B–C bond upon nucleophilic addition is largely dependent on the nature of the nucleophile. A unique feature of acyl boron species is that the tetrahedral intermediate generated in several of the described methodologies does not necessarily lead to loss of the B–C bond *via* a bora-Brook rearrangement.^21^ This contrasts the known reactivity of acyl metalloids, such as acylsilanes, which are reported to undergo the Brook rearrangement during processes such as the condensation with amines.^22^ The absence of this mode of reactivity in acylboron species is surprising based on the thermodynamic preference of boron to reside on heteroatoms, such as oxygen, and can likely be attributed to the tetracoordinate state of boron, which halts unwanted migration.

**Scheme 2:**
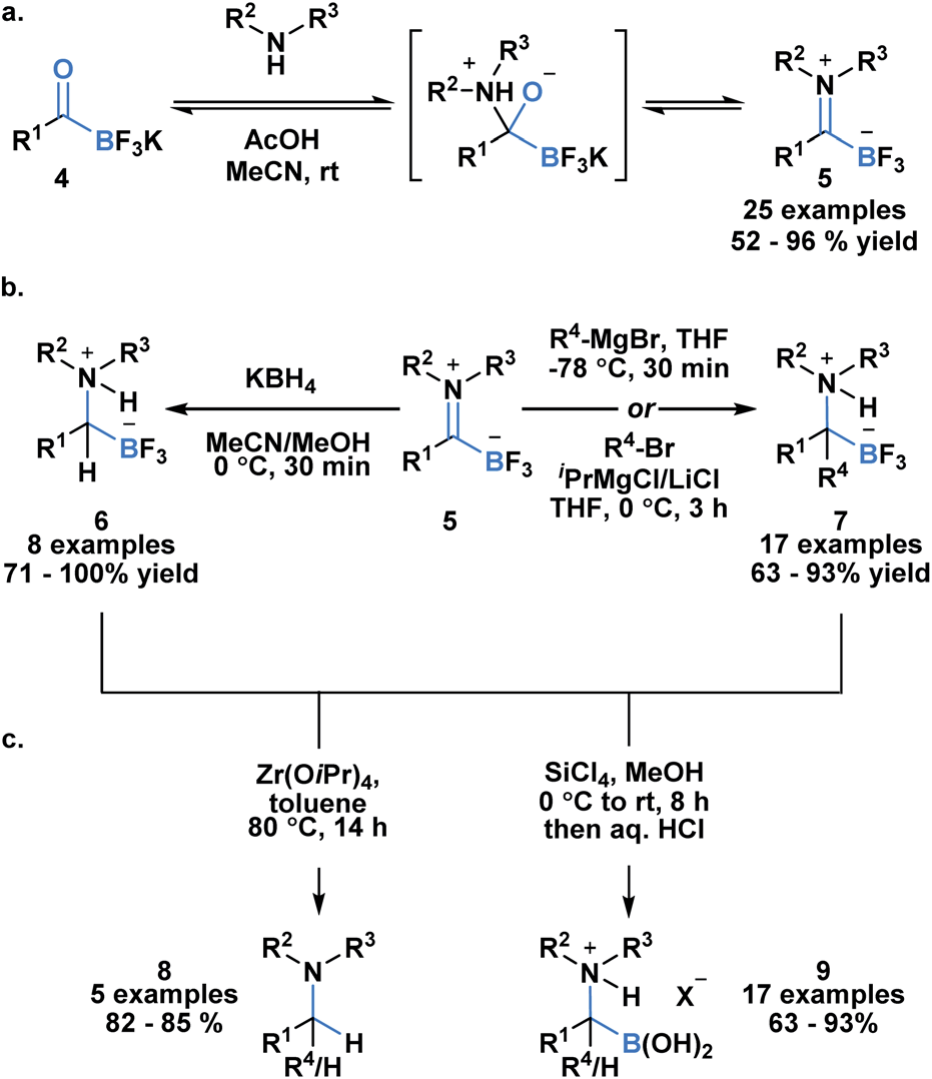
(a) Acylboron compounds are amenable to condensation with amines, giving access to the synthetically useful intermediate borylated iminium; (b) trifluoroborate iminiums can be transformed into α-aminotrifluoroborates either through reduction or Grignard addition; (c) subsequent protodeborylation or hydrolysis provides tertiary amines or α-aminoboronic acids.

Acylboron species have been reported to undergo condensation-driven processes to generate products such as borylated iminium species and heterocycles.^17,19,23–25^ Recently, Bode *et al.* demonstrated that air, moisture and chromatographically stable trifluoroborate iminiums (TIMs) **5** could be isolated in high yields from KATs using acidic conditions in polar solvent (Scheme 2a).^26^ The corresponding TIMs can be further transformed into α-aminotrifluoroborates through reduction with KBH_4_ (**6**) or addition with Grignard reagents (**7**) (Scheme 2b). Subsequent protodeborylation of α-amino-trifluoroborates with Zr(O*i*Pr) produces tertiary amines **8** in excellent yields (Scheme 2c). Alternatively, defluorination with SiCl_4_ leads to the generation of α-aminoboronic acids **9**, which are not easily prepared with current methodology.^27,28^

**Scheme 3:**
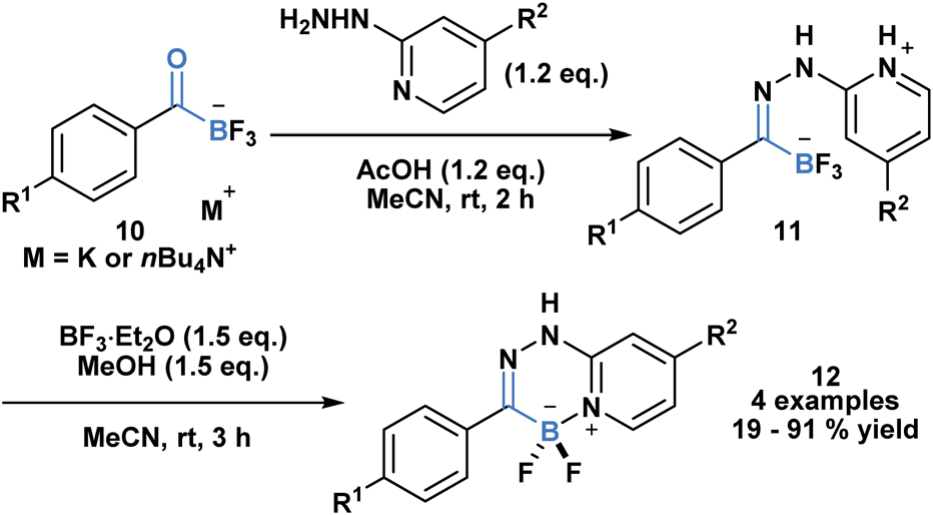
Hydrazone **11** generated from KAT **10** and 2-hydrazinopyridine can undergo Lewis acid mediated cyclization to generate a new class of C,N-chelated borates (**12**) that exhibit luminescence.

The high propensity of KATs to form imines was utilized by Ito and coworkers to develop a new class of boronate luminophores (Scheme 3).^29^ Specifically, KATs **10** were condensed with 2-hydrazinopyridines (**11**) and subjected to a BF_3_·Et_2_O mediated defluorocyclization between boron and the pyridine nitrogen. The resulting C,N-chelated borates **12** were stable to column chromatography and exhibited substituent dependent luminescence in the solid state.

**Scheme 4:**
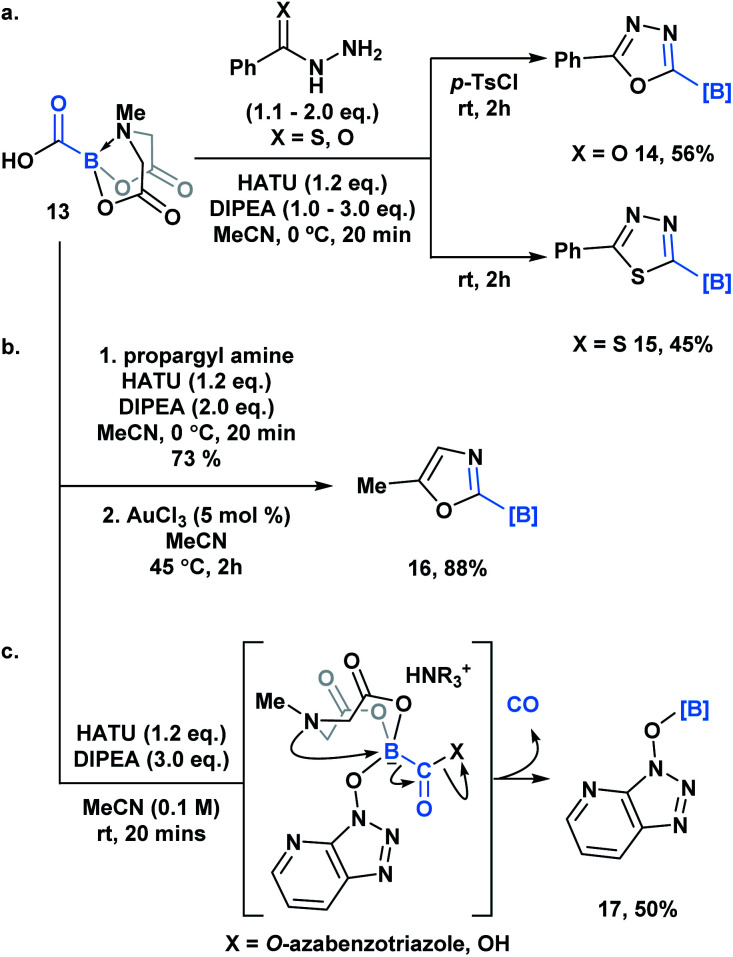
(a) One-pot, two-step approach to generate borylated 1,3,4-oxadiazoles **14** and borylated thiadiazole **15** from carboxyboronate; (b) Ag-catalyzed formation of 2-borylated 1,3-oxazole **16** from propargyl amide; (c) observation of *O*-borylated azabenzotriazole (**17**) by-product suggests a C-to-O migration of the hemilabile BMIDA group, which is coupled with CO release.

Yudin and coworkers recently demonstrated the synthesis of the C1 building block, carboxy-MIDA-boronate (Scheme 4, **13**), which can undergo reactions characteristic of organic carboxylic acids, such as HATU-mediated amide coupling reactions.^30*a*^ Similar to the previous examples, formation of the tetrahedral intermediate in this context does not lead to unwanted bora-Brook migration products, thereby enabling the synthesis of boron-containing products, such as carbamoyl-boronates, which are stable to column chromatography. **13** and the corresponding carbamoylboronates are amenable to cyclization processes generating a variety of difficult to access borylated heterocycles. For example, 1,3,4-oxadiazole **14** and 1,3,4-thiadiazole **15** were accessed through a one-pot condensation of the *in situ* generated hydrazide (Scheme 4a), while oxazole **16** was synthesized through a gold-mediated cyclization of propargyl carbamoylboronate (Scheme 4b).

Unexpectedly, during the optimization of the HATU-mediated amide coupling reaction, Yudin and coworkers reported the generation of *O*-borylated azabenzatriazole **17**, which through GC-TCD analysis was determined to proceed with release of carbon monoxide gas (Scheme 4c). It was hypothesized that *O*-azabenzotriazole might mediate B–N displacement, promoting scission of the B–C bond and the generation of the thermodynamically favored B–O bond. Another plausible mechanism might involve a 1,2-bora-Brook type rearrangement of the carbonyl bound *O*-azabenzotriazole *via* a three-membered transition state.^14,21,31^**13** was also found to release CO upon heating. Based on its tolerance of various reactive functionality, **13** been utilized as an *in situ* CO surrogate in various Pd-catalyzed carbonylative transformations.^30*b*^

**Scheme 5:**
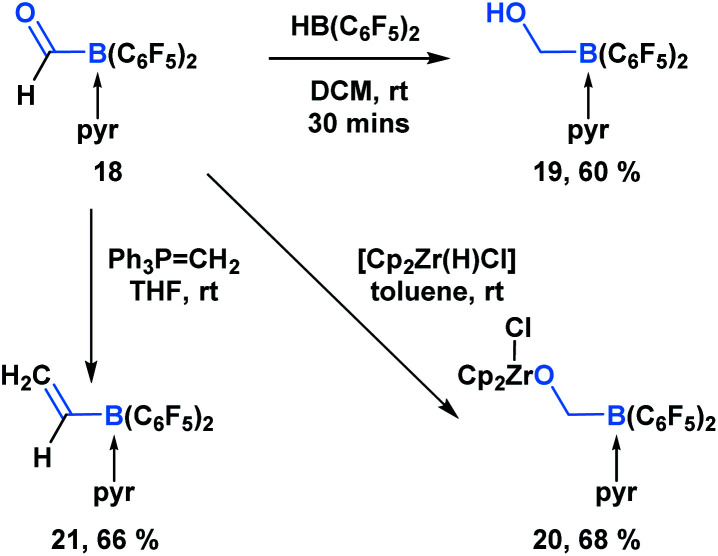
Pyridine-stabilized formyl borane **18** can be transformed into the corresponding reduction and olefination products in good yields.

In addition to KATs and MIDA boronates, pyridine-stabilized formyl borane can undergo processes that transform the carbonyl functionality but leave the C–B bond intact (Scheme 5).^19^ Erker and coworkers demonstrated that the reduction of **18** with HB(C_6_F_5_)_2_ led to the formation of boryl methanol **19** in 60% yield. Reaction with the Schwartz reagent produced the reduction product **20** in 68% yield. Additionally, the Wittig olefination product **21** could be obtained in 66% yield through the reaction with phosphorus ylide Ph_3_P=CH_2_.

**Scheme 6:**
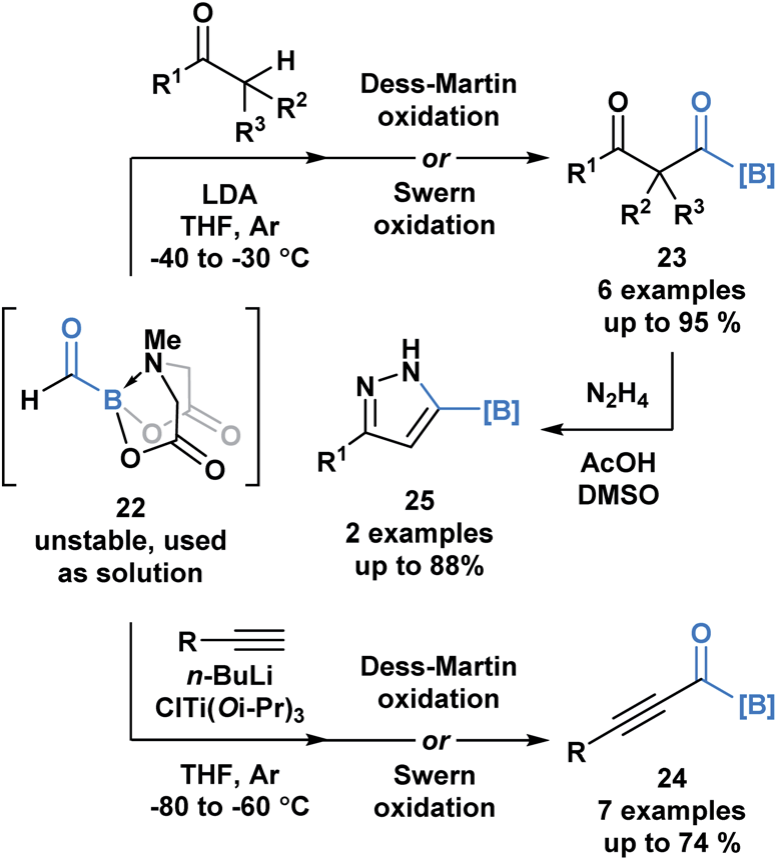
One-pot reaction sequences of formyl MIDA boronate **22** with carbon-based nucleophiles, followed by oxidation provides modular access to various acylboron compounds.

A recent report by Grygorenko and coworkers described the *in situ* synthesis of formyl MIDA boronate **22** through the Swern oxidation of hydroxymethyl MIDA boronate.^32^ The stability of **22** depends on the concentration of its solution and therefore, **22** was utilized as a solution in one-pot reaction sequences with various nucleophiles (Scheme 6). Subjection of the resulting addition products to oxidation conditions provided access to β-dicarbonyl MIDA boronates **23** and alkynyl acylboronates **24**. The synthetic potential of **23** was emphasized through the condensation-driven formation of pyrazole MIDA boronate **25**.

**Scheme 7:**
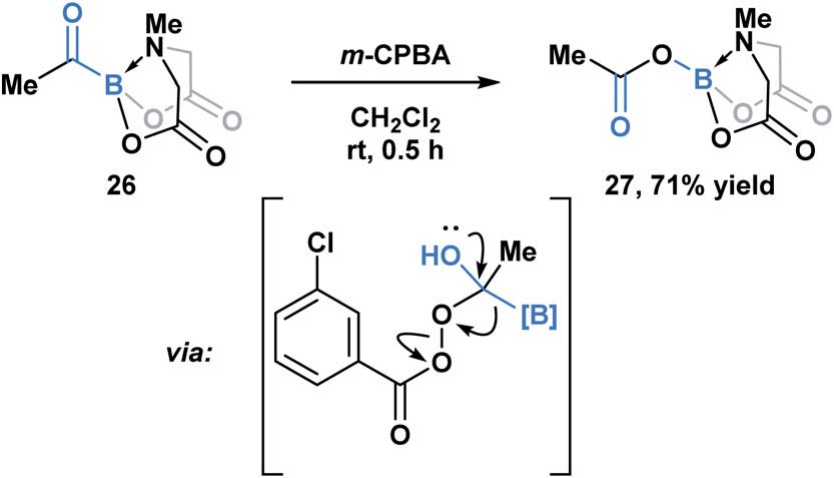
Propensity of MIDA boron to migrate was demonstrated in the Baeyer–Villiger oxidation of acyl boronate **26**.

**Scheme 8:**
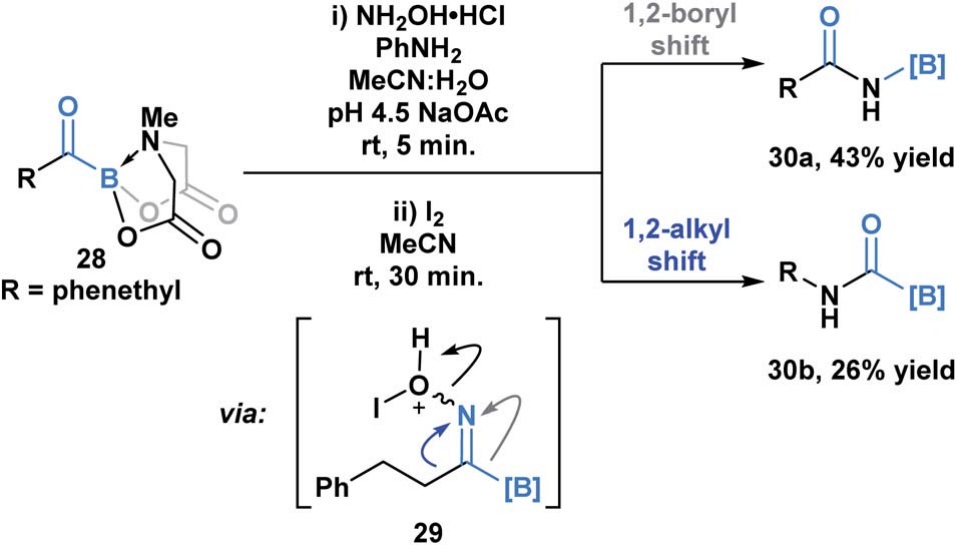
Iodine-mediated Beckmann rearrangement leads to both boryl (**30a**) and alkyl (**30b**) migration products.

Yudin and coworkers have demonstrated that the B–C bond of MIDA acylboronates can be cleaved in the context of a variety of rearrangement processes (Scheme 7).^33^ A Baeyer–Villiger oxidation of acylboronate **26** with *m*CPBA led to the generation of boryl carboxylate **27***via* nucleophilic addition of the peroxyacid onto the carbonyl carbon followed by exclusive boron migration onto oxygen. In addition, the Beckmann rearrangement of *in situ* generated boryl ketoxime **29** led to the formation of *N*-borylated amide **30a** (Scheme 8). Since the selectivity of the reaction is a consequence of the *E*/*Z* configuration of the oxime, such that the group *anti* to the leaving group is the one that migrates, the generation of carbamoylboronate **30b** was also observed.

Another unique feature of acylboron species is their capacity to serve as a surrogate of the carbonyl functionality in amide forming processes. Since Molander's pioneering demonstration of the amide ligation between KATs and azides in 2010,^15^ a variety of acyl boron species have been successfully applied in this context.^34–36^ Overall, these processes typically eliminate the necessity for auxiliary activating reagents and are highly chemoselective, making them suitable candidates for bio-conjugation methodologies.

**Scheme 9:**
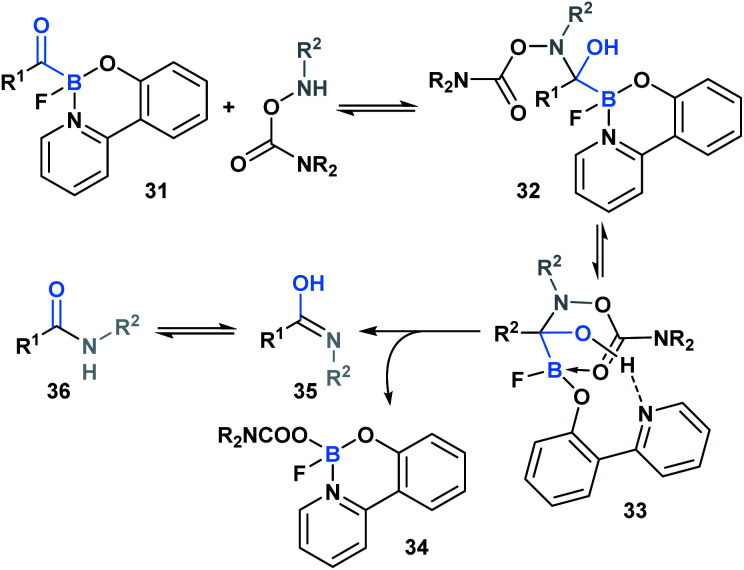
Ligation between *O*-carbamoylhydroxylamines and monofluoroacylboronates proceeds *via* hemiaminal formation, pyridine dissociation/carboxyl association and elimination *via* a six-membered transition state.

In 2015, Bode and coworkers showed that the reaction between *O*-carbamoylhydroxylamines and monofluoroacyl-boronates (**31**) led to the formation of an amide bond (Scheme 9).^36^ A mechanism was proposed involving initial attack of the hydroxyl amine nitrogen at the carbonyl carbon to generate hemiaminal **32**, which then underwent a concerted elimination of the B–C and N–O bonds. This was further supported through a DFT-mediated study conducted by the Fu group, who also determined that hemiaminal formation is followed by pyridine ligand dissociation and carboxylate coordination with the boron center to generate **33**. Subsequent elimination *via* a six-membered-ring transition state, and final water-assisted tautomerization generates the corresponding amide (**36**).^37^ The utility of this ligation strategy was emphasized through the development of a traceless, templated amide forming process that proceeds at low micromolar concentrations in aqueous media.^38^

**Scheme 10:**
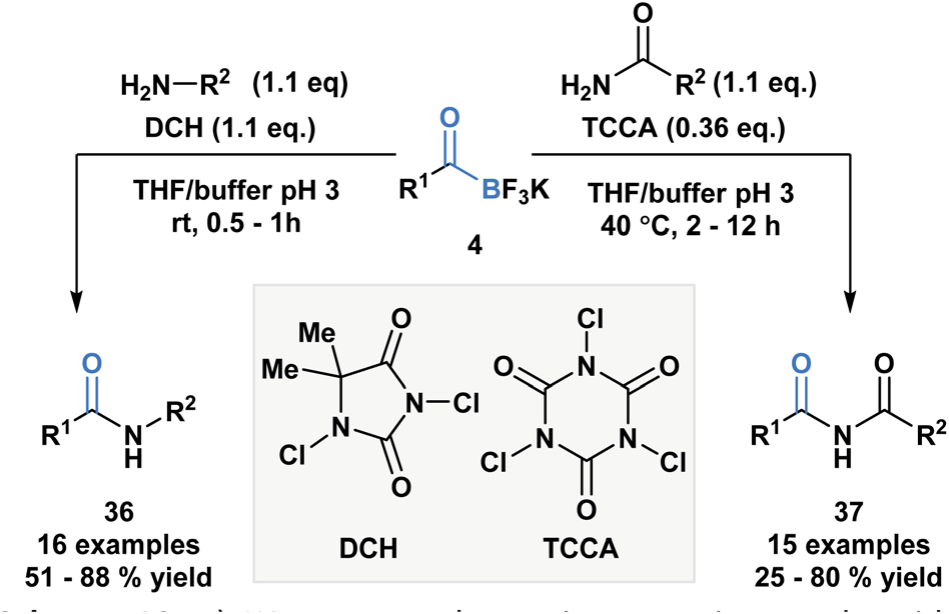
KATs can acylate primary amines and amides using inexpensive chlorinating agents, **DCH** and **TCCA**.

Bode and coworkers also demonstrated an amide formation process between primary amines or amides with KATs (**4**) promoted by simple chlorinating agents, such as 1,3-dichloro-5,5-dimethylhydantoin (**DCH**) and 1,3,5-trichloroisocyanuric acid (**TCCA**) (Scheme 10).^36^ The process is highly chemo-selective and tolerant of a broad range of functional groups that lead to the desired amide (**36**) and imide (**37**) products in moderate to excellent yields. Mechanistically, the researchers favor direct interaction of the *in situ* generated *N*-chloroamine and the KAT carbonyl group over interaction of the nucleophile with the boron atom, followed by acyl migration.

**Scheme 11:**
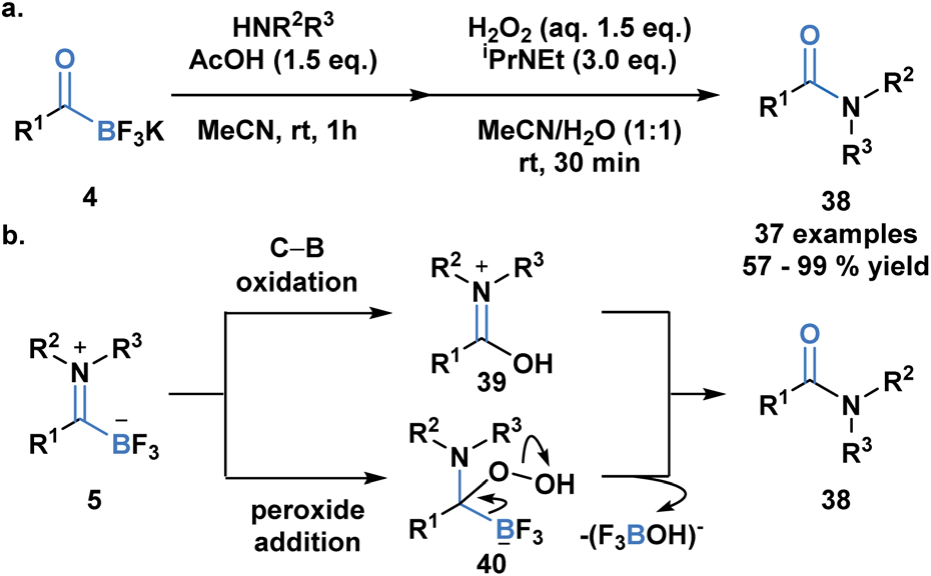
(a) The oxidative amidation of KATs enables the synthesis of tertiary and secondary amides *via* TIMs, without the need for coupling reagents; (b) possible mechanisms for oxidative amide formation from TIM; mechanistic insights support peroxide addition as the more likely reaction path.

Previously developed ligation reactions were limited to the synthesis of secondary amides. To address this limitation, Bode *et al.* developed an oxidative amidation from KATs (**4**) or TIMs (**5**) under aqueous conditions, which enable access to both secondary and tertiary amides (**38**) (Scheme 11a).^39^ The absence of problematic coupling reagents makes this process a viable alternative to classical amide forming reactions and a promising method to selectively functionalize peptides and proteins. The authors considered two mechanistic possibilities for the transformation, involving either direct B–C bond oxidation (**39**) or peroxide addition into the carbonyl carbon (**40**) (Scheme 11b). Based on several observations and supporting literature, the latter mechanism was deemed the more plausible reaction path.

## Silicon

**Fig. 4:**
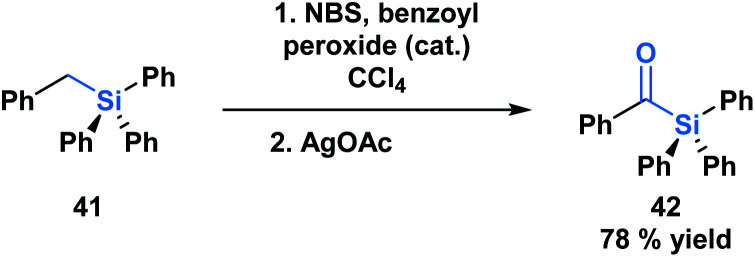
The first synthesis of an acylsilane reported by Brook.

The first acylsilane – triphenylsilyl ketone (**42**) – was synthesized in 1957 by Brook in an endeavour to explore the α-silicon effect (Fig. 4a).^40^ The synthesis involved an oxidative bromination with *N*-bromosuccinimide (NBS) and subsequent Ag(i)-assisted hydrolysis of benzyltriphenyl-silane **41**. In this report, Brook noted several key properties of the acylsilane, such as a carbonyl infrared stretch that was comparable to acetophenone (∼1600 cm^−1^), the color and rapid decomposition under basic conditions. These findings inspired a flurry of research led by Brook, ultimately establishing the diverse reactivity profile of acylsilanes, which includes the Brook rearrangement and the photolytic formation of α-siloxy-carbenes *via* heterolytic or homolytic cleavage of the C–Si bond, respectively.^41^ These and other modes of reactivity available to acylsilanes can be attributed to several factors – the oxophilicity and fluorophilicity of silicon, the potential for hypervalency on silicon and the photolytic reactivity of acylsilanes.^42^ The application of acylsilanes has led to the development of highly enabling transformations under mild conditions that have been recognized and discussed broadly by several instructive reviews – both in terms of reactivity and synthesis of acylsilanes.^43,44^

**Scheme 12:**
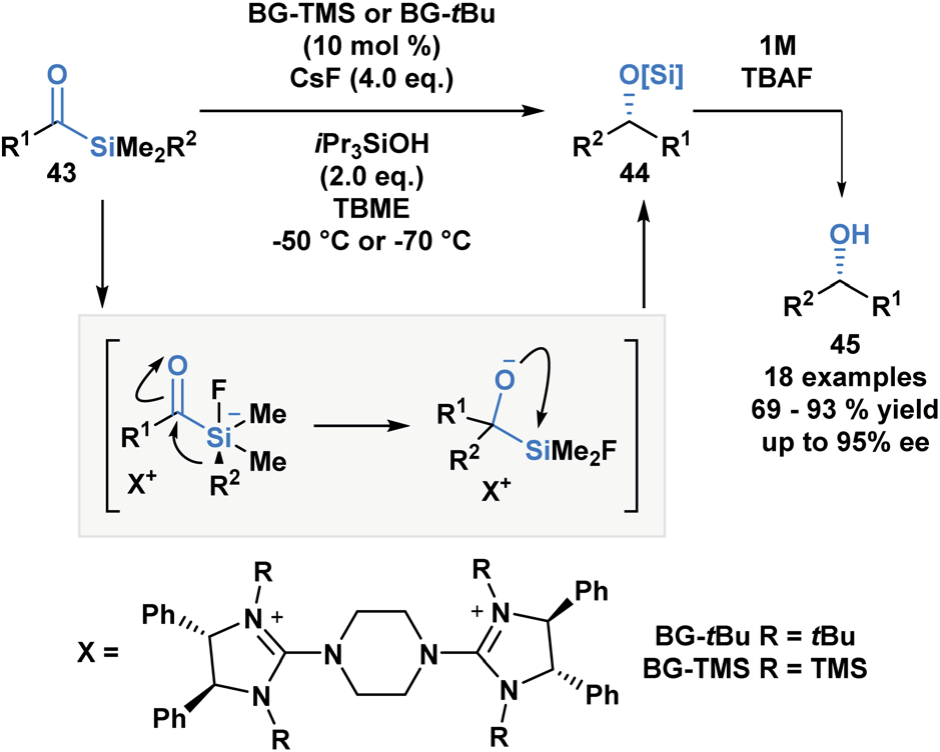
The asymmetric synthesis of secondary alcohols from acylsilanes through a tandem 1,2-anionotropic/Brook rearrangement. TBME = *tert*-butyl methyl ether.

A large proportion of acylsilane reactivity is governed by mechanisms initiated by nucleophilic addition to the carbonyl carbon. Tan and coworkers leveraged this reactivity to develop a highly enantioselective synthesis of secondary alcohols (Scheme 12).^45^ Specifically, silicon's fluorophilicity and potential hypervalency was utilized to affect a 1,2-anionotropic shift that was directed by a chiral bis-guanidinium (BG) cation. This was followed by a [1,2]-Brook rearrangement and a tetrabutylammonium fluoride (TBAF) – mediated desilylation, generating the alcohols in moderate to excellent yields, with high enantioselectivity (**45**). In this case, the migrating groups were limited to aryl and allylic substituents, and electron-deficient aryl moieties resulted in faster migrations due to the increased electrophilicity of the silicon centre.

**Scheme 13:**
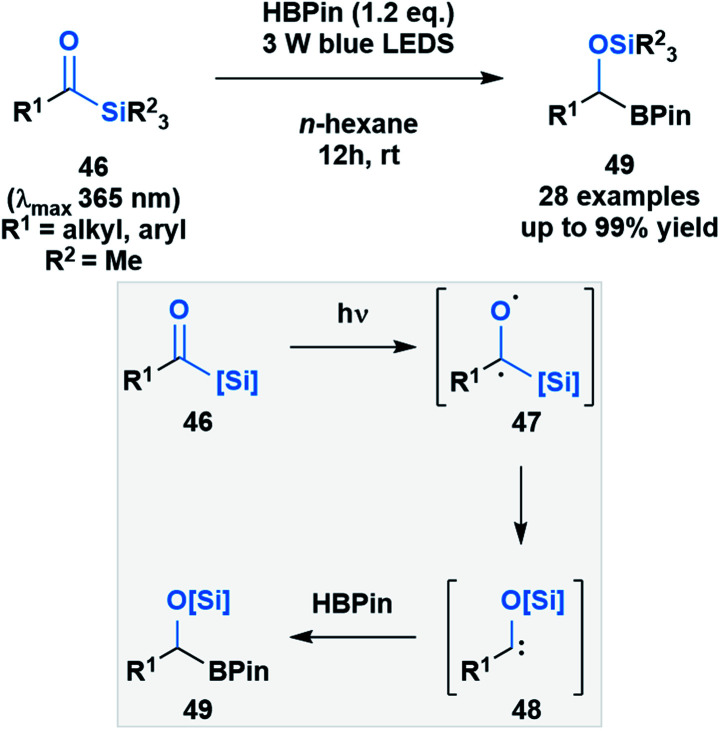
B–H insertion of photochemically generated α-siloxycarbenes yields α-boryl alcohols in quantitative yields (top); proposed formation of α-siloxycarbenes and its reaction with HBpin (bottom).

Brook noted that acylsilanes have a strong absorption in the near-UV and visible range that is attributed to an n→π* transition. Excitation *via* this transition can trigger a photolytic cleavage that leads to the generation of α-siloxy-carbenes.^41^ The propensity of these species to undergo insertion chemistry has been known since their initial discovery,^46^ but was recently exploited by Glorius and co-workers to generate silyl-protected α-boryl alcohols **49** (Scheme 13).^47^ The mechanism of the reaction was elucidated by DFT calculations, which showed the formation of an intermediate triplet diradical species (**47**) that undergoes an α-elimination, followed by a 1,2-silyl shift to afford the corresponding α-siloxycarbene (**48**). The B–H insertion step was proposed to go through a concerted mechanism based on comparative DFT calculations of the two potential pathways.

**Scheme 14:**
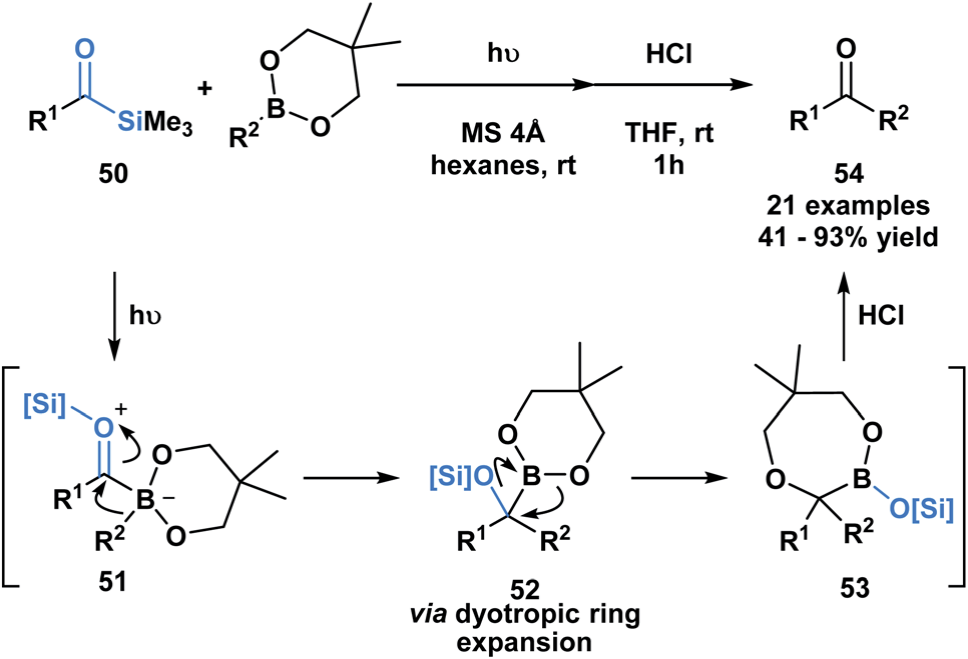
Photochemically promoted coupling of acylsilanes and organoboronic esters delivers unsymmetrical ketones through a formal B–C bond insertion. MS = molecular sieves.

Kusama and co-workers showcased an unusual example of the formal insertion of an α-siloxycarbene into a B–C bond (Scheme 14).^48^ They reported a method to generate ketones **54** through the coupling of acylsilane **50** and an organoboronic ester. As opposed to the example reported by Glorious, which involved a concerted insertion, the authors propose that the α-siloxycarbene initially interacts with the empty p-orbital of the boronic ester to generate a zwitterionic boronate **51**, that undergoes an alkyl migration generating **52**.^48^ A dyotropic rearrangement of **52** yields the ring expanded silyl boronic ester **53**, which upon mild acid hydrolysis by silica gel affords the corresponding ketone. This mechanism is supported by NMR characterization of the intermediates **52** and **53**.

**Scheme 15:**
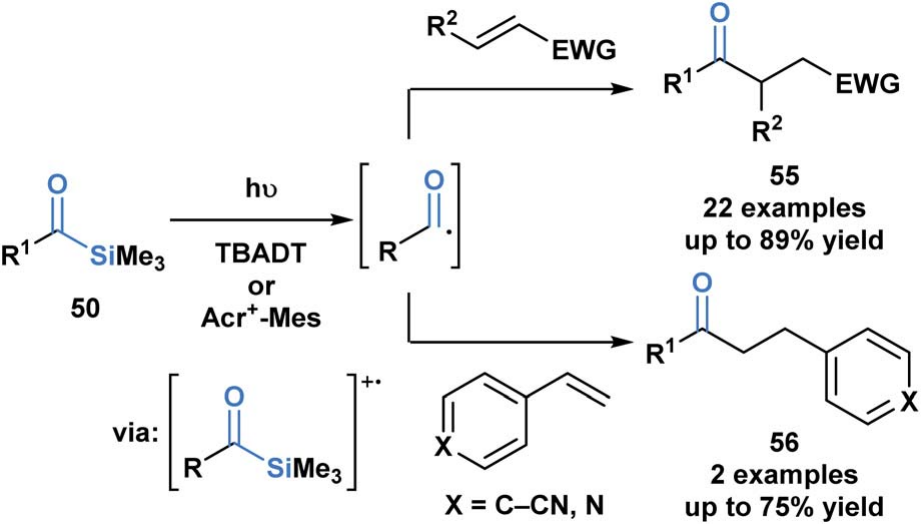
Acyl radicals generated from acylsilanes under photoredox reaction conditions underwent Giese addition yielding unsymmetrical ketones. Acr^+^–Mes = 9-mesityl-10-methylacridinium, TBADT = tetra-*n*-butylammonium decatung-state.

Fagnoni and coworkers demonstrated that the photochemical reactivity of acylsilanes could be directed towards the selective formation of acyl radicals, as opposed to α-siloxycarbenes, through an acridinium or tungstate-sensitized photoredox cycle (Scheme 15).^49^ The acyl radicals generated were subsequently trapped *via* a Giese addition with a range of activated olefins to afford ketone products **55** and **56**. This report represents a fundamentally novel discovery in the photochemistry of acylsilanes.^46^

**Scheme 16:**
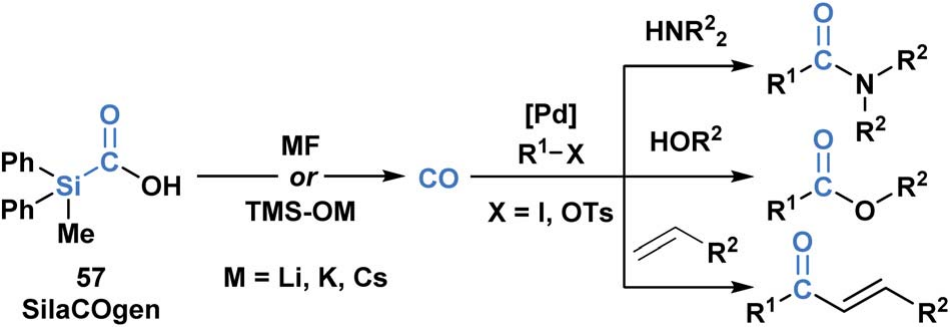
Decarbonylation of SilaCOgen can be promoted *via* a range of nucleophiles. The generated CO has been harnessed in various carbonylative coupling processes.

Skrydstrup and co-workers reported the synthesis of a series of silacarboxylic acids to develop an air-stable and easy to handle CO-releasing molecule (CORM).^50*a*^ Previous work established that high temperatures or basic conditions were necessary to promote CO extrusion from silacarboxylic acids, which limited their utility in applications such as carbonylative coupling chemistry.^51^ It was found that by subjecting silacarboxylic acids to fluoride or oxygen-based nucleophiles, near quantitative CO release could be achieved. Mechanistically, it is proposed that the nucleophiles might directly interact with silicon and mitigate the corresponding fragmentation, or alternatively, could deprotonate the acid and promote a 1,2-Brook rearrangement. SilCOgen (**57**), which was found to be most efficient, has been utilized as both an *ex situ* or *in situ* source of CO for a variety of Pd-catalysed carbonylative couplings (Scheme 16).^50^ The CO-releasing capacity displayed by **57** resembles that of carboxyboronate **13**.^29^

**Scheme 17:**
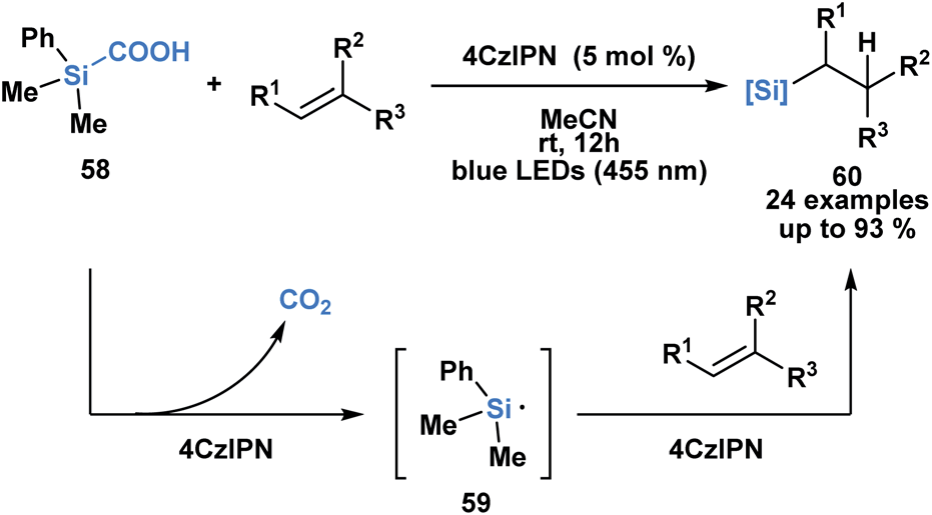
Silacarboxylic acids as precursors to silyl radicals through visible light photocatalysis. 4CzIPN = 1,2,3,5-tetrakis-(carbazol-9-yl)-4,6-dicyanobenzene.

Uchiyama and co-workers envisioned silacarboxylic acids as potential precursors for synthetically viable silicon-centred radicals through a photochemical decarboxylation (Scheme 17).^52^ Previously, silyl radicals were primarily generated from silanes through H-atom transfer. The authors found that under conditions of indirect photoexcitation using a carbazole-based photosensitizer, 4CzIPN, the homolytic cleavage of the C–Si bond of **58** yielded a silyl radical **59**. A subsequent Giese-type addition to trisubstituted olefins afforded functionalized alkylsilanes **60**. Similar reactivity was observed with germa-carboxylic acids using an iridium-based photosensitizer.

**Scheme 18:**
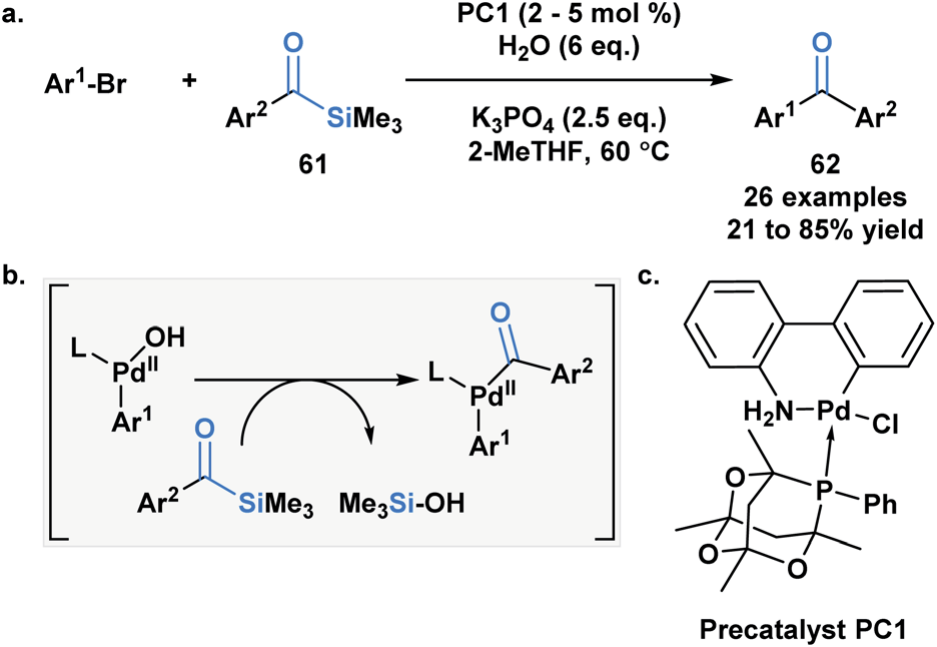
Cross-coupling of aroylsilanes with aryl and hetero-aryl bromides.

Schmink and Krska showcased a reversed-polarity approach towards the synthesis of unsymmetrical diaryl ketones **62** utilizing acylsilanes (**61**) as an acyl anion equivalent.^53^ This was accomplished using a palladacycle pre-catalyst with a bulky phosphaadamantyl ligand. Mechanistically, **61** is proposed to undergo transmetallation with a palladium hydroxo intermediate, followed by reductive elimination to afford the desired diaryl ketone product (Scheme 18). Currently, acylsilanes are the only acyl metalloid species that undergo transmetallation chemistry at the carbon-metalloid bond.

**Scheme 19:**
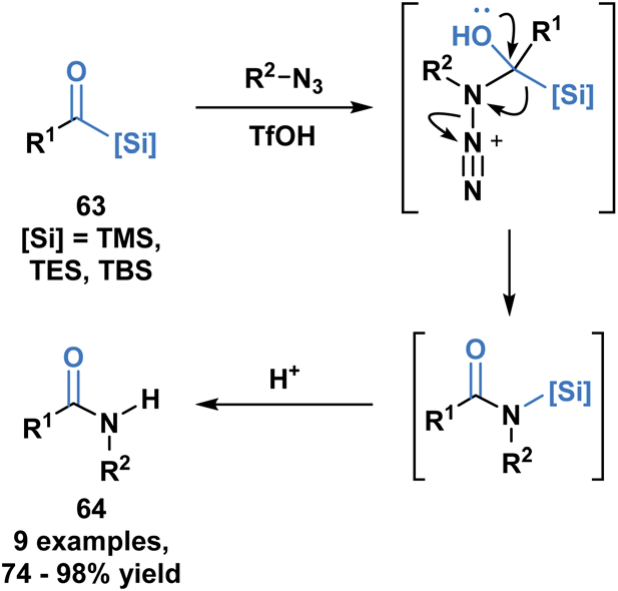
Synthesis of secondary amides through acid-catalyzed coupling of acylsilanes and alkyl azides.

Another example of the heterolytic cleavage of the C–Si bond in acylsilanes was demonstrated in an Aube–Schmidt reaction, wherein the silicon moiety showed a greater migratory propensity as compared to an alkyl group (Scheme 19).^54^ The treatment of aroylsilanes (**63**) with alkyl azides in the presence of triflic acid afforded secondary amides (**64**) in excellent yields. While this is an unusual method towards the formation of an amide bond, the tetrahedral intermediate generated could serve to inspire reactivity involving silicon migration reactions.

**Scheme 20:**
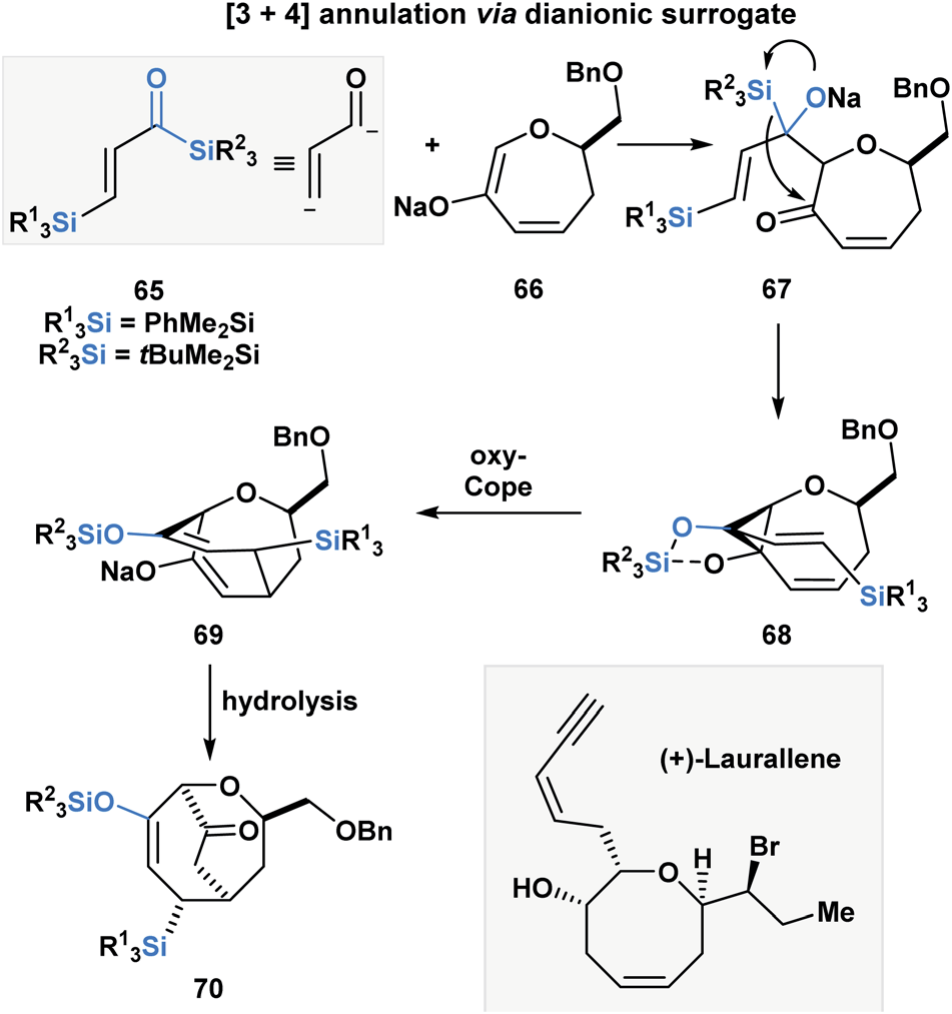
Formal total synthesis of (+)-laurallene – towards the Crimmins' intermediate.

Incorporating acylsilanes within the route towards a target of total synthesis has allowed for several enabling synthetic strategies. In the formal total synthesis of (+)-laurallene (Scheme 20), Takeda utilized vinylogous acylsilane **65** to effect a [3 + 4]-annulation with **66** through a tandem Brook rearrangement/oxy-Cope sequence.^55^ The desilylated α,β-unsaturated ketone substrate is composed of two acylsilanes – a direct and a vinylogous acylsilane, and reacts as a masked dianion. The initial Brook rearrangement is triggered by the 1,2-addition of enolate **66**. The masked carbanion **67** then adds into the carbonyl to form the cyclopropylalkoxydiene **68**, which then undergoes an oxy-Cope to furnish the [3.3.2]-bicyclic system **70** upon hydrolysis. **70** was further elaborated into the Crimmins' intermediate, which can be converted into (+)-laurallene.

**Scheme 21:**
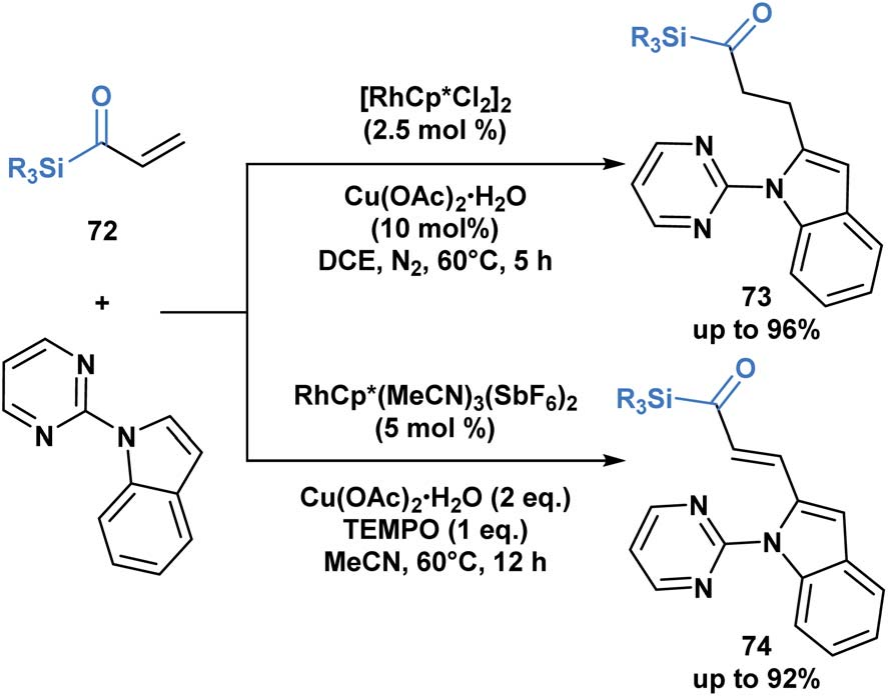
Divergent hydroarylation and cross-coupling of acryloyl silanes by rhodium-catalyzed C–H activation retains the C–Si bond. Cp* = 1,2,3,4,5-pentamethylcyclopentadienyl anion.

The previous examples depict acylsilanes as a compound class capable of reacting under several reaction conditions, typically resulting in the cleavage of the C–Si bond. Loh and co-workers have showcased a set of conditions under which acryloyl silanes **72** are arylated at the β-position while preserving the C–Si bond (Scheme 21).^56^ Divergent reactivity is obtained by tuning the reaction conditions to deliver either alkylated (**73**) or alkenylated (**74**) indole products. This represents a mild method towards introducing the acylsilane functionality into indole scaffolds.

## Germanium

**Fig. 5:**
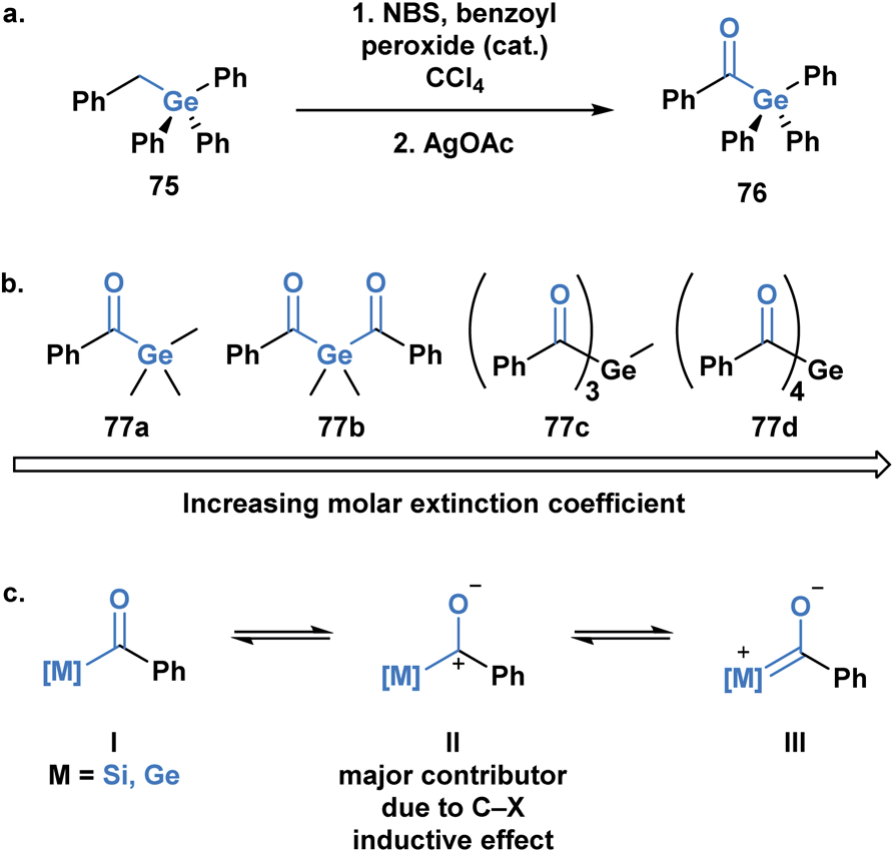
(a) First reported acylgermane; (b) resonance contributions in acylsilanes and acylgermanes; (c) trend observed in the molar extinction coefficients of mono-, bis-, tris- and tetra-acylgermanes.

Germanium was once termed *eca*-silicon by Mendeleev at a time when the periodic table was being developed.^57^ In the same manner, acylgermanes were first synthesized by Brook through a synthetic route analogous to acylsilanes (Fig. 5a).^58^ The first acylgermane, benzoyl(triphenyl)germane **76**, was observed to have similar spectral and structural properties to acylsilanes, such as a highly redshifted ultraviolet absorption band of the carbonyl group due to a strong n→π* (d_π_–p_π_) interaction.^59^ The corresponding IR stretch (∼1625 cm^−1^) suggests the carbonyl character of this class, which is further supported by a C=O bond length of 1.20 Å; the C–Ge single bond is reported as 2.01 Å, longer than the previous acylmetalloids.^60^

For several decades after the first synthesis, acylgermanes were solely studied as close analogs of acylsilanes and showed very little in terms of divergent reactivity, however, a germanium-based variant of the Brook rearrangement has not been reported to date. The reactivity of acylgermanes is dominated by photochemical processes, whereas polar reactions are considerably less reported. Similar to acylsilanes, the basis for the unique polar reactivity of acylgermanes compared to their carbon analogues is rooted in the high contribution of the polarized resonance structure **II** (Fig. 5b). In 1966, Yates and Agolini ran comparative pK_BH_ measurements on ketones, acylsilanes and acylgermanes, determining that the acyl-metalloids were significantly more basic than the ketones. Further molecular orbital calculations showed a negligible C–O π-bond character in both cases providing evidence for the significant contribution of the charge separated resonance structure.^61^

The development of acylgermanes as visible-light photoinitiators by the Liska group in 2008 marked a resurgence in the field.^60^ In 2018, Gescheidt and coworkers performed a comparative study of the photoinitiating capabilities of mono- to tetra-acylgermanes, including the synthesis of the first tris-acylgermane **77c** (Fig. 5b).^62^ Acylgermanes are the only class of acyl metalloids with polyacyl metalloid variants. An increase in molar extinction coefficient was observed with additional acyl groups, which was attributed to the enhanced conjugation of the system (Fig. 5c). Poly(acylgermanes) have not been used in synthetic applications but represent a curious class of stable molecules, notably different from their silicon counterparts, of which only bis(acylsilanes) are known.^63^

**Scheme 22:**
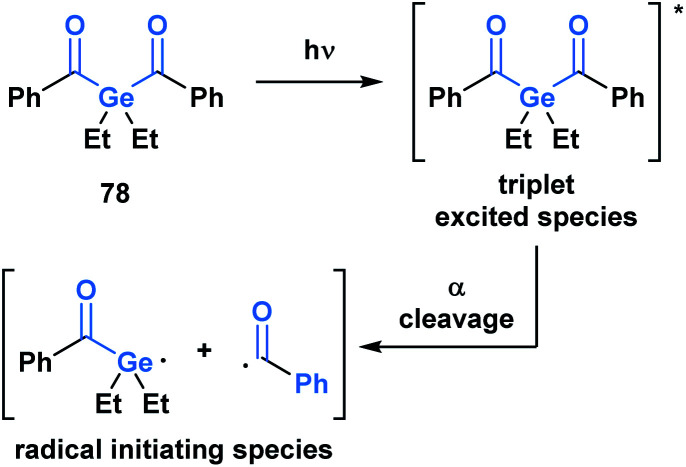
Development of acylgermanes as visible-light photoinitiators for radical polymerization in dental filling material and artificial tissues.

Upon irradiation by an appropriate light source, acylgermanes are photoexcited to a triplet-state species, which then undergoes an α-cleavage to afford a germanium-centred radical and a benzoyl radical that serve as radical initiators (Scheme 22).^64^ Mono- and bis-acylgermanes (such as **78**) were developed as visible-light alternatives to acylphosphine oxide based photo-initiators. Owing to a decreased toxicity profile, acylgermanes have become promising candidates for photoinitiators for polymerizations in biomedical applications such as dental fillings and artificial tissue materials.^65^

**Scheme 23:**
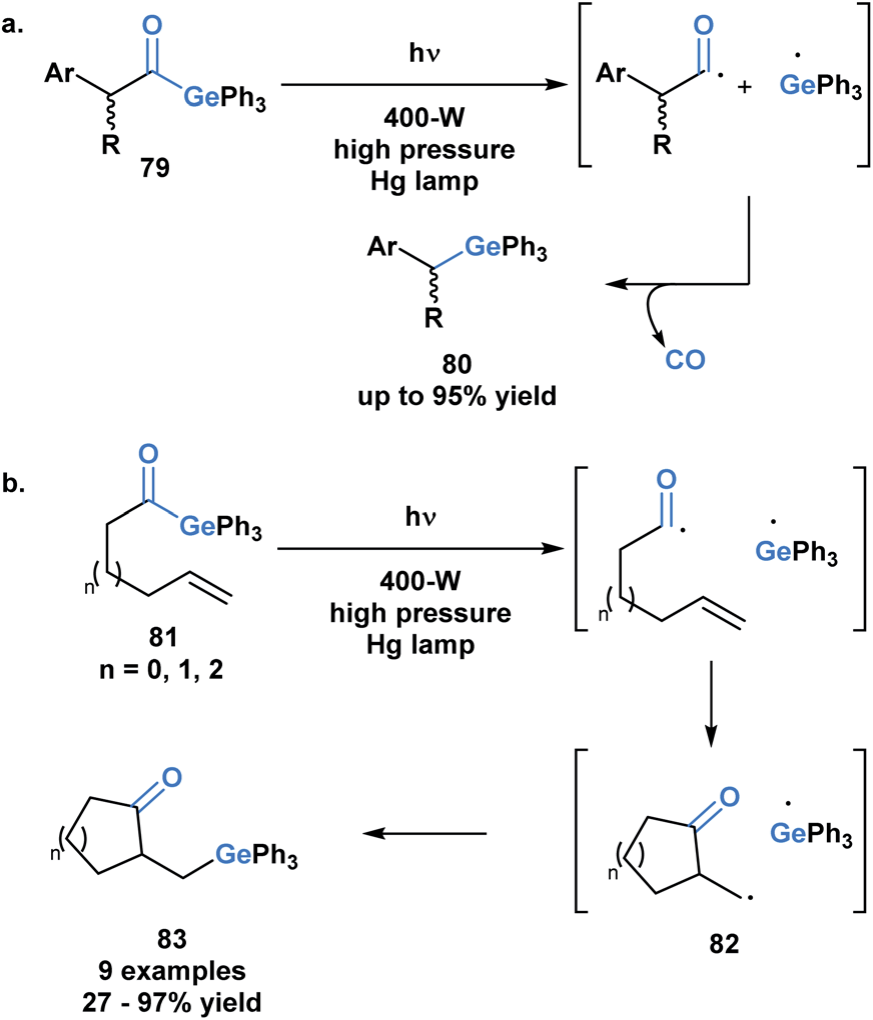
(a) UV irradiation of (α-arylacyl)triphenylgermanes leads to decarbonylation and generation of (α-arylalkyl)-triphenylgermanes; (b) photolytic generation of acyl and germyl radicals resulting in a 5-*exo-trig* radical cyclization followed by radical recombination.

The use of benzoylgermanes is well suited to photoinitiator applications since it results in stable benzoyl and germyl radicals with long half-lives allowing for efficient photoinitiation. An alternate fate for the photolytically-generated radicals was observed by Kiyooka and coworkers (Scheme 23a).^66*a*^ Under UV irradiation, acylgermane **79** was homolytically split into an acyl radical-germyl radical pair. Following decarbonylation, radical recombination afforded (α-arylalkyl)triphenylgermane **80**. In a related study, Kiyooka and coworkers showed that the acyl radical-germyl radical pair generated from alkenyl acylgermane **81** could undergo a stepwise addition across an alkene in an *exo-trig* manner to form **82**. This intermediate subsequently undergos a radical recombination to form germyl ketone **83** (Scheme 23b).^66*b*^ Curran and coworkers expanded on this reactivity and contrasted it with that of acylsilanes under similar conditions.^66*c*^

**Scheme 24:**
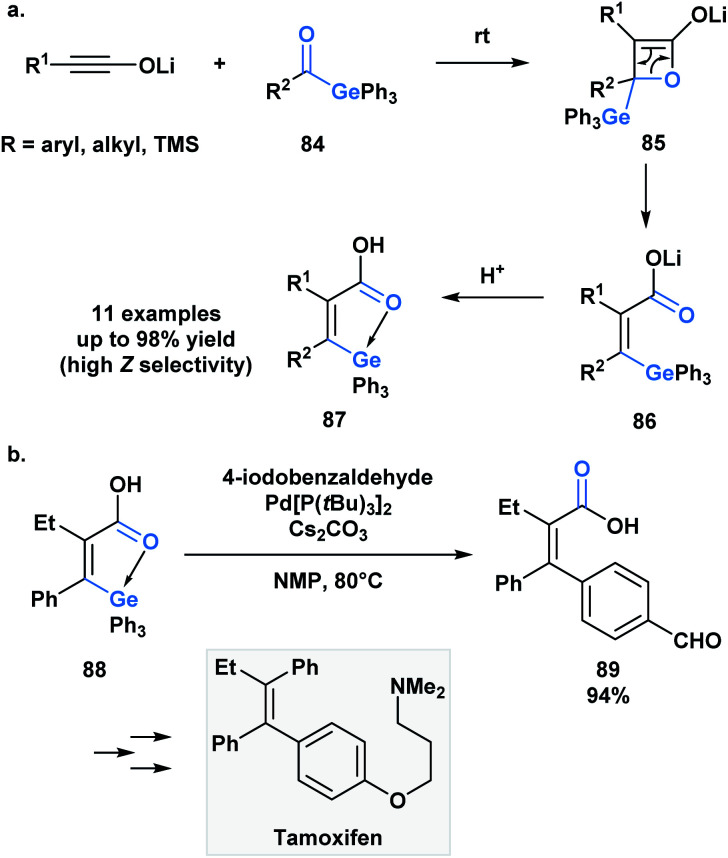
(a) Germanium-directed torquoselective ring opening of β-lactone enolates; (b) palladium-catalyzed cross-coupling of intramolecularly activated alkenylgermane was reported and applied towards the stereoselective synthesis of (*Z*)-tamoxifen.

As in the case of silicon, the capacity of germanium to be hypervalent can play a guiding role during a reaction. As an extension to a research program investigating torque-selective ring openings of β-lactone enolates derived from ketones, Shindo and co-workers synthesized multi-substituted alkenyl germanes **87** in a highly *Z*-selective manner from lithium ynolates and acylgermanes **84** (Scheme 24a).^67^ The β-lactone enolate intermediate **85** underwent a torquoselective electrocyclic ring opening directed by the low-lying σ* of the Ge–C bond. Aromatic acylgermanes resulted in higher *Z*-selectivity than aliphatic acylgermanes because of the greater π-electron donating nature of aromatic substituents, which results in a preference for the germyl substituent to rotate outwards during ring opening. Similar torquoselectivity was observed with acylsilanes through an analogous orbital interaction.^68^

Based on this initial report, Shindo and coworkers were interested in exploiting the intrinsic coordination of the alkenylgermanes in the context of a palladium-catalyzed cross-coupling reaction (Scheme 21b).^69^ Interestingly, the authors found that the coupling reaction between alkenylgermanes and aryl iodides was more efficient than the related alkenylsilanes. The utility of this method was emphasized by the stereoselective synthesis of tamoxifen.

**Scheme 25:**
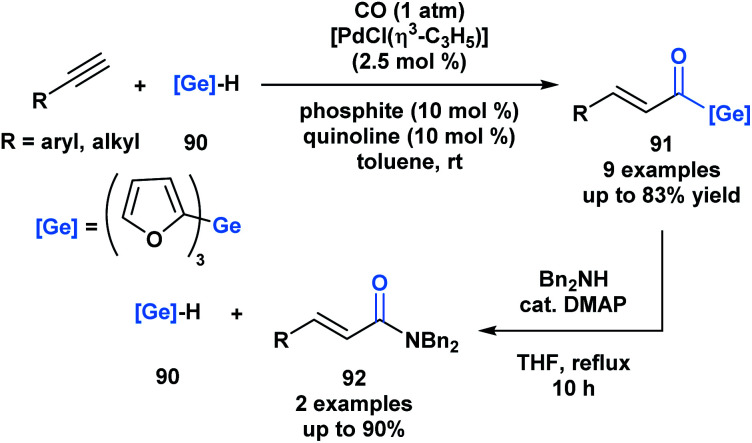
Hydrogermacarbonylation of alkynes leads to the generation of α,β-unsaturated acylgermanes, which can be further converted into amides through an addition–elimination sequence.

Oshima and coworkers reported the first example of a hydrogermacarbonylation of an alkyne to generate α,β-unsaturated acylgermanes **91** (Scheme 25).^70^ The reaction conditions were compatible with various functional groups, such as hydroxyl and amine groups. Internal alkynes were unsuitable substrates. Within the same pot, the generated α,β-unsaturated acylgermanes were found to react with amines to afford amides (**92**) and the corresponding (trifuryl)germane. Acylsilanes under similar conditions are converted into α-aminocarbinols through the Brook rearrangement instead of the elimination of silane.^21^ This is a consequence of the lower oxophilicity of germanium, and its greater ability to stabilize a negative charge due to its higher polarizability.

## Tellurium

**Scheme 26:**
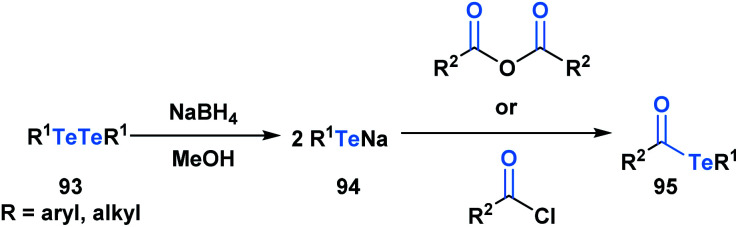
Reaction of ditellurides with acyl chlorides or acid anhydrides yields the corresponding acyl metalloid.

Acyl tellurides, or telluroesters, represent a class with promising synthetic utility and several comprehensive reviews have been published portraying their synthesis and applications.^71–73^ There are various methods to synthesize acyl tellurides (**95**), but the most broadly applied approach involves the *in situ* reduction of ditellurides (**93**) with borohydride to generate telluride anions (**94**), which are subsequently trapped with acyl chlorides or anhydrides (Scheme 26).^74–77^ Akin to the previous acyl metalloids, IR and ^13^C NMR spectroscopy of various acyl tellurides suggest their carbonyl-like character. This is further supported by X-ray crystallographic analysis,^78^ which reveals bond lengths that are indicative of a C=O double (1.20 Å) and C–Te single bond (2.15 Å). In comparison to acyl boron species, acyl tellurides are not known to undergo condensation-driven processes at the carbonyl carbon. Early attempts to generate the corresponding tellurium-containing hydrazine by reacting acyl telluride with *p*-nitro-phenylhydrazine or phenylhydrazine led to no observable reaction in the former case, and generation of diaryl ditelluride in the latter.^75^ Instead, the reactivity of acyl tellurides is dominated by processes that directly cleave the C–Te bond, proceeding by either polar or radical mechanisms. The radical reactivity, which can be accessed through thermal or photocatalyzed conditions, has become increasingly popular in the last decade, particularly in the context of natural product synthesis.

**Scheme 27:**
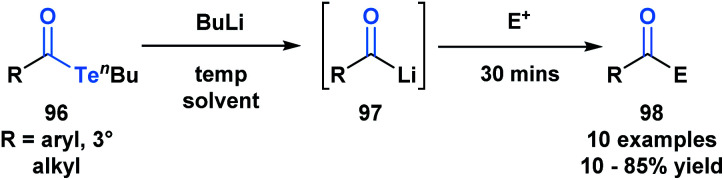
Lithium–telluride exchange of acyl tellurides **96** leads to acyllithium species **97**, which can further react with various electrophiles.

Sonoda and coworkers have demonstrated the ability of acyl tellurides to function as surrogates of acyl anions.^79^ They identified that acyl tellurides (**96**) not bearing an α-hydrogen underwent a lithium-telluride exchange to generate a variety of acyllithiums (**97**), which further reacted with electrophiles, such as ketones, aldehydes or silanes (**98**) (Scheme 27). This transformation was found to be ineffective with the corresponding selenoesters and acylstannanes. The success of this processes might be rationalized by the high reactivity of organotellurides towards alkyllithiums. This method is practically useful given the stability and accessibility of **96** in comparison to the kinetically reactive acyl lithium species.

**Scheme 28:**
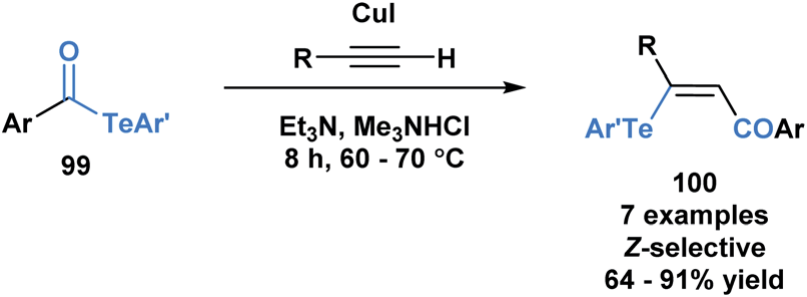
Telluroacylation of terminal alkynes provides access to (*Z*)-β-(aryltelluro)-α,β-unsaturated ketones.

Another application of acyl tellurides was reported by Wang and co-workers.^80^ They demonstrated that they could access (*Z*)-β-(aryltelluro)-α,β-unsaturated ketones (**100**) through a telluroacylation of terminal alkynes in the presence of CuI and triethylamine (Scheme 28).^81^ In the presence of cuprous ions, it was previously demonstrated that telluroesters can be attacked at the carbonyl carbon by various nucleophiles, such as water and alcohols.^82^ In this paper, the authors propose that the *in situ* generated acetylide can attack the carbonyl carbon of acyl telluride (**99**), expelling telluroarene and producing an intermediary alkynone. The corresponding arenetellurol then undergoes 1,4-addition to the alkynone, generating the observed α,β-unsaturated ketone.

**Fig. 6:**
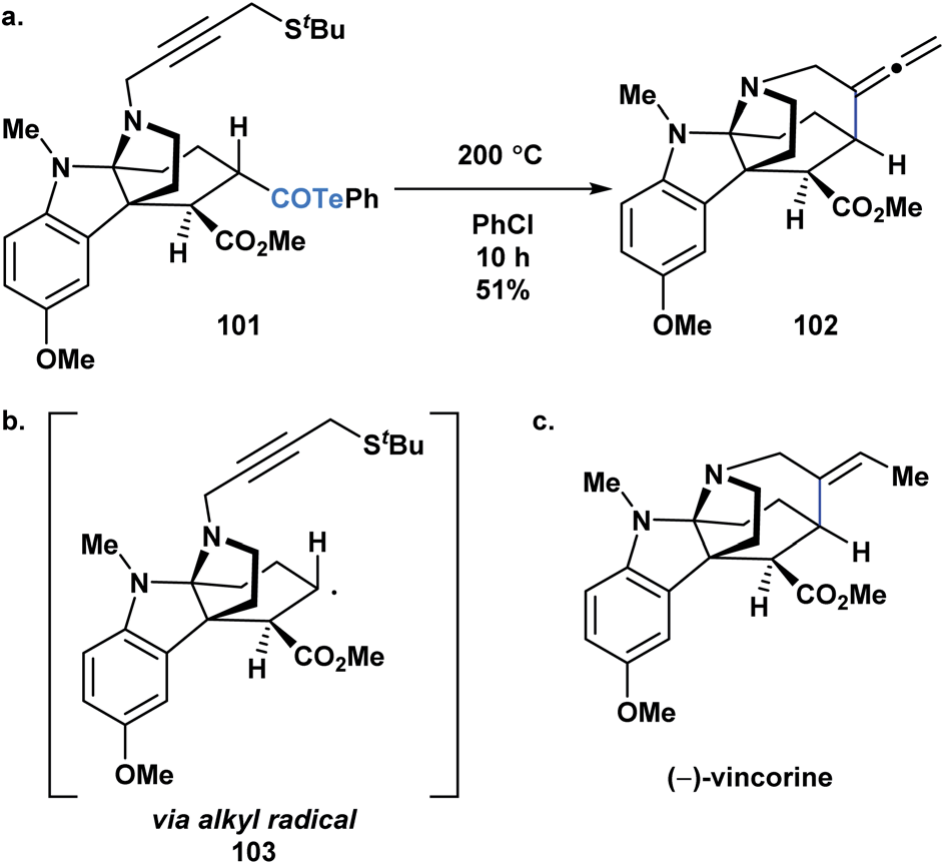
(a) A key step in the synthesis of (−)-vincorine involves a radical cyclization of acyl telluride precursor **101**; (b) the reaction was postulated to proceed *via* generation of the corresponding alkyl radical (**103**); (c) structure of (−)-vincorine.

Crich and coworkers established that acyl tellurides can serve as sources of acyl radicals upon photolysis with a 250 W tungsten lamp or thermal activation (benzene at reflux).^76,83,84^ The generated acyl radicals are reactive towards radical trapping agents, such as TEMPO, diphenyl diselenide, diphenyl disulphide and *N-t*-butyl-α-phenylnitrone, as well as amenable to intramolecular cyclization/tellurium transfer processes. Yamago, Yoshida and coworkers reported that at elevated temperature, acyl tellurides can undergo a radical-mediated group transfer with isonitriles, generating α-acyl substituted imidoyl tellurides in good to high yields.^85^ In 2013, MacMillan and coworkers applied the radical chemistry of acyl tellurides to generate the challenging seven-membered azepanyl ring system in (−)-vincorine (Fig. 6c).^86^ Therein, the researchers evaluated various radical precursors, such as the Barton ester and selenoesters, but ultimately found that upon thermal initiation, the acyl telluride precursor (**101**) generated the desired azepanyl allene (**102**) most efficiently. Mechanistically, it was postulated that the transformation occurred *via* C–Te bond homolysis, followed by decarbonylation to furnish an alkyl radical (**103**) (Fig. 6b), which could then undergo the 7-*exo*-dig cyclization to install the desired ring (Fig. 6a). This report was the first example of acyl tellurides functioning as an alkyl radical precursor.

**Scheme 29:**
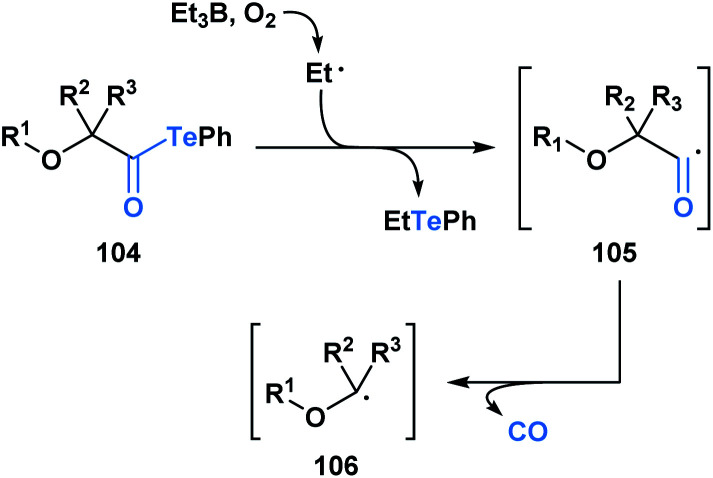
Et_3_B-mediated radical decarbonylation of α-alkoxyacyl tellurides leads to the generation of α-alkoxy radicals, which are useful synthetic intermediates in the synthesis of polyoxygenated products.

Since 2015, Inoue and co-workers have made significant contributions towards advancing the synthetic application of acyl tellurides, especially towards the assembly of densely oxygenated products. Specifically, several mild radical-based coupling reactions of α-alkoxyacyl tellurides **104**, which are readily derived from monosaccharides, have been developed.^87^ In the coupling reactions, an ethyl radical generated from Et_3_B/O_2_ (ref. 88) promotes C–Te bond homolysis, leading to the formation of acyl radical **105** (Scheme 29). The favorable interaction between the lone pair of the adjacent oxygen and the σ* of the intermediate acyl radical facilitates C–CO bond scission, leading to the generation of α-alkoxy radical **106**.

**Scheme 30:**
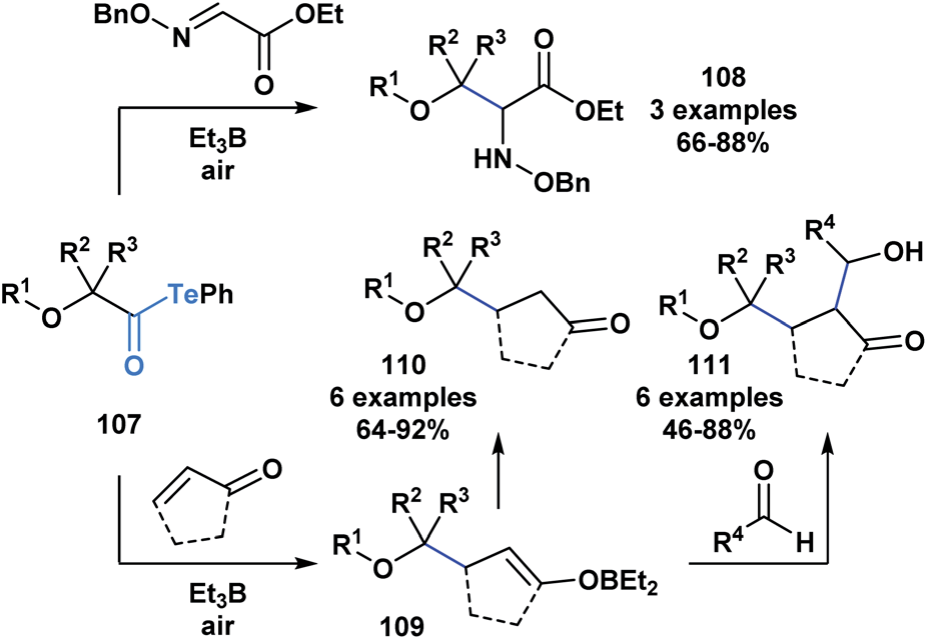
The reaction of α-alkoxyacyl tellurides with glyoxylic oxime ethers or enones generates two-component products **108** and **110**, respectively; the intermediate boron enolate **109** generated during the two-component processes can further react with an aldehyde to yield three-component adducts **111**.

The resulting α-alkoxy radical species were shown to intermolecularly react with glyoxylic oxime ethers (**108**) or enones (**110**) to generate two-component adducts (Scheme 30).^87^ Furthermore, through a radical polar mechanism, the intermediate boron enolate (**109**) generated during this process could be engaged in an intermolecular aldol reaction with various aldehydes, leading to the controlled generation of substrates with four contiguous stereocenters. The polyol structures (**111**) accessed through this approach are difficult to obtain by other methods.

**Scheme 31:**
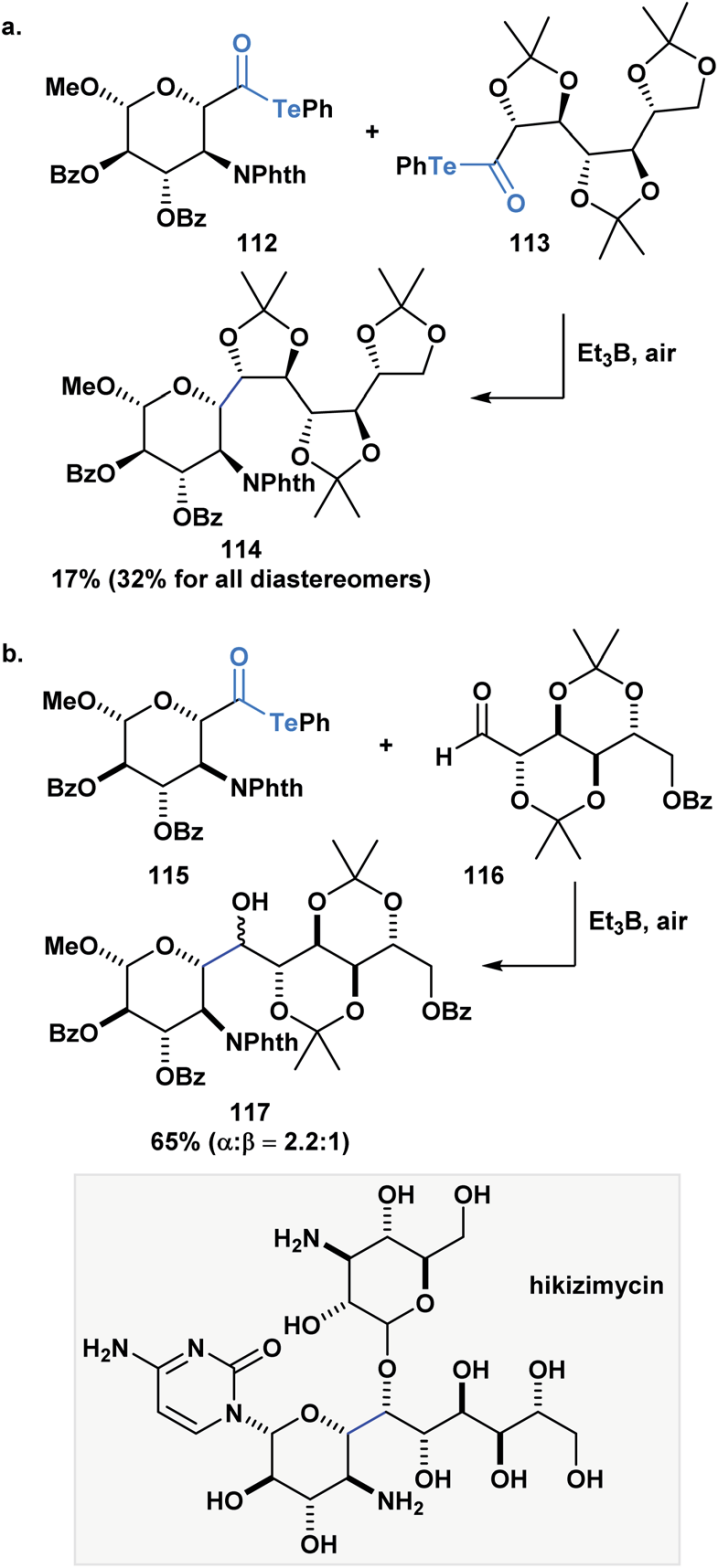
(a) The synthesis of the hikizimycin core can be accomplished in one step through a radical–radical cross-coupling reaction between two sugar-derived α-alkoxyacyl tellurides; (b) key step in the convergent synthesis of hikizimycin involves an intermolecular radical addition to an aldehyde.

Inoue and co-workers have also accomplished homo- and heterocoupling reactions of radicals generated from α-alkoxyacyl tellurides. The potential of this methodology was exemplified through the construction of the core (**114**) of hikizymicin, an antibiotic and antihelmintic agent, in a single and convergent step from d-galactose- (**112**) and d-mannose-derived monomers (**113**) (Scheme 31a).^89^ This endeavour represents a formidable challenge due to the increased synthetic complexity of cross-coupling radicals. Astonishingly, the 9 contiguous stereocenters of the hikizymicin structure were selectively built among the possible six homo- and three other hetero-coupled products.

More recently, Inoue and co-workers reported the convergent total synthesis of hikizimycin utilizing an unprecedented intermolecular addition of an α-alkoxy radical generated from acyl telluride **115** to aldehyde **116** as a key step (Scheme 31b).^90^ Radical additions to aldehydes can be problematic due to competing β-scission that reverses the reaction,^91,92^ but the addition of the α-alkoxy radical was found to deliver the corresponding adduct in a combined yield of epimers in 65%. The protecting groups incorporated in either fragment played an important role in favoring the formation of the desired **117**-α-isomer. Overall, these reports highlight the potential use of acyl tellurides to streamline the synthesis of polyoxygenated carbon chains, which are well-represented motifs in several natural products.

## Conclusions

Since Brook's pioneering work in the 1950s, the chemistry of acyl metalloids has greatly progressed beyond fundamental curiosity to attractive applications in modern organic synthesis. By combining the rich chemistry of carbonyls and metalloids, chemists have been presented with new opportunities to address various research problems. For example, the ability to selectively manipulate the carbonyl carbon of acylboron species, while retaining the B–C bond, allows the construction of otherwise difficult to access boron-containing frameworks. This mode of reactivity is inaccessible to other acyl metalloids and can be attributed to the protecting group around boron that attenuates its inherent electrophilicity. In contrast, engaging acylboron compounds with certain nucleophiles can lead to B–C bond cleavage, leading to boron migration or its complete removal. The latter has enabled the use of acylborons as carbonyl surrogates in amide forming ligation chemistry. In comparison, the applications of acylsilanes include both polar and radical transformations. The polar modes are accessed by engaging the carbonyl carbon or the silicon atom with various nucleophiles and often involve the well-known [1,2]-Brook rearrangement. Photolytic cleavage of the C–Si bond can lead to either the formation of carbene or radical intermediates, which have been creatively utilized in organic synthesis. The chemistry of acylgermanes was developed in the shadow of acylsilanes but lacks their characteristic polar reactivity. Instead, the photolytic cleavage of the C–Ge bond dictates the reactivity and applications of acylgermanes. In a similar fashion, applications of acyl tellurides are predominantly exploited in the realm of radical chemistry, with sparse examples of polar reactivity. The homolytic cleavage of C–Te has led to the formation of both acyl and alkyl radicals, wherein the latter has proven useful towards the synthesis of natural products.

The chemistry of acyl metalloids represents a continuously expanding field with exciting opportunities to develop novel transformations. Efforts into discovering new and taming existing reactivity in this space will undoubtedly lead to the identification of highly enabling processes.

## Author contributions

A. H. developed and assembled the sections on acylboron and acyl tellurides. C. N. A. developed and assembled the sections on acylsilanes and acylgermanes. A. H., C. N. A. and A. K. Y. wrote and edited the manuscript.

## Conflicts of interest

There are no conflicts to declare.
